# Progress in Medicine

**Published:** 1896-01-25

**Authors:** 


### Progress in Medicine.

### DISEASES OF THE LUNGS AND PLEURÆ.

(*Continued from page* 235.)

**Climatic Treatment.**—At Luxor there are comfortable hotels, and even as far south as Assouan good accommodation is afforded. These places are reached either by dahabeah, or post boat, or steamer. While the latter are more rapid in transit, the advantage of a quiet life on board a comfortable native boat or "dahabeah" will be appreciated by invalids.

E. Symes Thompson,[16] writing on the same subject, remarks that English people must learn the canon that recovery from chronic illness can only be secured by persistent, well-directed effort. An inferior place well adjusted produces better results than the best place misapplied. As regards Egypt, more depends on time and place than with other health resorts. First, as to the time of arrival. With all its attractions, charms, and engrossments, Cairo is not wholesome till mid-November; but the voyage out is then apt to be rough and trying to invalids, whereas it is often smooth in October. Referring to Helouan, he says that its sulphur springs are mainly in use for rheumatic patients wintering in Egypt. Some take the waters there a few times a week, going in and out of Cairo; but the excitements of Cairo are apt to tax their strength. The pure air, early hours, and complete repose of desert life are much more conducive to the success of the water treatment. Cairo is not suited to invalids, and every year's experience shows it to be terribly harmful to many who are tempted to remain there. The subsoil is always saturated during the floods; much of the residential quarter is built on midden heaps, and is percolated with cesspool soakage. Newcomers feel the steamy heat much, suffer much from neuralgia, and are liable to dengue fever.

As regards cases of phthisis, benefit is equally to be looked for in early and advanced cases, provided the conditions are not acute. The soothing influence exerted on the mucous membrane is of service alike in chronic bronchial affections with emphysema and in tuberculous conditions of the lung, where it is necessary to secure a pure, dry, warm atmosphere. If the acute phase has passed away, benefit may be expected in the first, second, or third stage of phthisis, especially where the lung affection has been pneumonic, bronchopneumonic, or pleuritic in origin. The value of the climate in convalescence from pulmonary disorders consequent on influenza, measles, enteric fever, &c., is very marked. Affections of the nasal, pharyngeal, and Eustachian membrane form a considerable proportion of the cases met with and treated with benefit in Egypt. The physician, Thompson remarks, when balancing the relative advantages of varying climates, must clearly decide whether the patient before him should bask lizard-like in the sun, or be stimulated to active exertion, whether he will be benefited by active exercise—*e.g.*, skating or toboganning—or by passive exercise on the deck of a dahabeah, or the back of a donkey. The Alps are for the young and the vigorous, the Nile for the old, the prematurely aged, and for those suffering from deteriorations and degenerations. When compared with the Riviera, Egypt is warmer, drier, and being practically rainless and cloudless, the amount of sunshine is greater. On comparing the climate of Egypt with that of the Mediterranean coast, the difference is found to be mainly due, in the one case, to the vicinity of the desert, from the other, to that of the sea. There are dangers and disadvantages in each, and it is a question of relative rather than absolute merit. More time can be spent in the open air in the Riviera or in Algiers than in any sunny haven on the English coast, and more time can be spent out of doors in Egypt than in either of the other countries just mentioned. When we compare Egypt with Madeira or the Canary Islands, we find that at Assouan the sunshine is more unbroken than even at Orotava or Las Palmas. Last winter the rainfall at Orotava was excessive; at Assouan it was *nil.*

These remarks on the nature of the climate of Egypt are very opportune, as many thousand visitors and invalids now go to this interesting land every year from the shores of England and America.

Another aspect of this important question is dealt with by Nahm,[17] the medical officer to the well-known sanatorium at Falkenstein. He has collected information from the death registers of the district with the object of seeing whether the deaths from phthisis amongst the inhabitants have been more numerous since the establishment of the institution and the consequent assemblage of consumptives in the locality. A table, extending from 1856 to 1894, which he has compiled, shows for each year the total number of inhabitants, the deaths from pulmonary tuberculosis, and those due to other causes. For twenty years before the establishment of the sanatorium the average death-rate (amongst the inhabitants) was 4·0 per 1,000 living, or 18·9 per cent. of all deaths; whereas, for eighteen years during which the sanatorium had existed, the average death-rate had fallen to 2·4 per 1,000 living, and to 11·9 per cent. of all deaths. Nahm considers that in the face of such figures the danger of the neighbourhood being infected by the erection of sanatoria for consumption is without justification.

**Bacteriology, and Pathology.**—The work of R. L. Straus[18] on tuberculosis and its bacillus will be read with interest, coming from one whose name is so intimately associated with advances in our knowledge of the pathology of tuberculosis. His statements on some moot points are especially noteworthy. It was Straus and Gameleïa who showed that the tubercle bacillus after death preserves in a large measure the pathogenic properties characteristic of the living microbe. The introduction of these dead bacilli into the circulation determines lesions which in every respect resemble those due to living cultures, showing that the power of determining tubercular lesions resides in a special substance contained in the dead bacilli. Thus, even if one could succeed in killing Koch's bacillus, recovery from tuberculosis is not therefore assured. Further, these investigations have shown that, contrary to what is usually believed, the cultures of the bacillus of avian tuberculosis differ from those of the bacillus of human or mammal tuberculosis, not only by certain bacteriological peculiarities, but also in their pathogenic effects. He therefore concluded that avian tuberculosis is distinct from human tuberculosis. He discusses some very important points bearing on the acquirement of the disease. Hereditary transmission of predisposition is unquestionable, as is also the fact of family contagion. Intra-uterine infection, determined by the passage of the bacillus through the placenta, has also been proved clinically and experimentally, but appears to be of exceptional occurrence. On the contrary, no proof has yet been brought forward of ovular infection, resulting from contamination of the ovule, before fecundation, by a phthisical mother. Ingesta, more particularly milk and meat from tuberculous animals, have also been accused of serving as vehicles for the tubercle bacillus. Without denying that infection may be possible by this means, Straus declares that he does not know of a single authenticated case in which tuberculosis has been transmitted to man by the ingestion of meat from tuberculous animals. Milk is known not to be dangerous unless the cow from which it is derived presents tubercular lesion of the udder. Rare as such accidents are, they may be effectually guarded against by using only thoroughly cooked meat and boiled milk. Another point is dealt with, viz., the assertion that the fever of consumptives is not due to Koch's bacillus, but to the penetration into the blood of staphylococci and streptococci. Straus dissents from this view, for an examination of blood,[19] taken directly from the veins of patients who had reached the hectic stage, has always yielded negative results to him. Further, it is a mistake to consider the microbes of suppuration as the agents of the pneumonic or broncho-pneumonic infiltration which supervenes in the cause of phthisis, Straus never having been able to detect these microbes in circum-tubercular exudations; and the pneumococcus is but rarely met with under these conditions.

Straus' remarks on avian tuberculosis are of importance when we remember the number of diseased domesticated birds with which many of the community are constantly associated. Delépine[20] writes on the prevalence of tuberculosis in domesticated animals. Cattle are, with man, the mammals most liable to the disease, about 16 per cent. being infected. Swine are not very often infected, but in the horse numerous instances have been put on record, although the animal is very generally supposed to be immune. In the sheep tuberculosis is undoubtedly rare, about 11 in 10,000 being the proportion furnished by Danish and German statistics. The goat is not immune; not only can it be infected experimentally, but several cases of non-experimental tuberculosis have been recorded, while instances of tuberculosis in the dog and cat are becoming more and more frequent. Regarding tuberculosis in birds, he says it is important not to be influenced too much by the differences between avian and human tuberculosis. We have to deal here with only varieties of the same bacillus. Such remarks are of great importance in view of the prevalent belief that the serum of some of these animals may be used for the cure of phthisis in man, none of the so-called immune animals proving to be so in reality in the light of Delépine's investigations.

[9] N. Y. Med. Journ., Aug. 3rd, 1895. [10] Montreal Med. Journ., Sept., 1895; Sp. B. M. 7, Oct. 26th, 1895. [11] Med. Week, Sept. 20th, 1895. [12] Med. Week 26, vii., 1895. [13] Lancet, Nov. 2nd, 1895. [14] Bristol Med. Chir. Journ., Dec., 1895. [15] Egypt as a Winter Resort, p. 25. [16] Practitioner, Dec., 1895. [17] Münich Med. Woch., Oct. 1st, 1895; Pract., Nov., 1895. [18] Review in Med. Week, July 19th, 1895. [19] Med. Week, 1895, pp. 265—268. [20] Med. Chron., Sept., 1895.

